# Draft Genome Sequence of Geobacter sp. Strain OR-1, an Arsenate-Respiring Bacterium Isolated from Japanese Paddy Soil

**DOI:** 10.1128/genomeA.01478-14

**Published:** 2015-01-29

**Authors:** Ayaka Ehara, Haruo Suzuki, Seigo Amachi

**Affiliations:** a Graduate School of Horticulture, Chiba University, Matsudo, Matsudo City, Chiba, Japan; b Graduate School of Science and Engineering, Yamaguchi University, Yoshida, Yamaguchi, Japan

## Abstract

Here, we report a draft genome sequence of Geobacter sp. strain OR-1, an arsenate-respiring bacterium isolated from Japanese paddy soil. It contained two distinct arsenic islands, one including genes for a respiratory arsenate reductase (Arr) as well as for arsenic resistance (*arsD-arsA-acr3-arsR-arrA-arrB*) and the second containing only genes for arsenic resistance.

## GENOME ANNOUNCEMENT

Arsenate-respiring bacteria may play an important role in arsenic release from anoxic sediments in the form of arsenite (1). Although respiratory arsenate reductase genes (*arrA*) closely related with Geobacter spp. have frequently been detected in arsenic-rich sediments (2, 3), it is still unclear whether they directly participate in arsenic release, mainly due to the lack of pure cultures capable of arsenate reduction. Previously, we isolated a novel arsenate-respiring bacterium, Geobacter sp. strain OR-1, from Japanese paddy soil (4). The strain utilized not only arsenate but also soluble Fe(III), ferrihydrite, nitrate, and fumarate as electron acceptors. To date, at least two Geobacter species, G. uraniireducens Rf4 and G. lovleyi SZ, are known to possess *arr* genes in their genomes, but arsenate respiration by these bacteria has not yet been reported. Thus, strain OR-1 is the first Geobacter species shown to be capable of arsenate respiration, and it is useful as a model microorganism that potentially impacts the mobilization of arsenic in flooded soils and anoxic sediments (4). Here, we report the draft genome sequence of strain OR-1.

Strain OR-1 was grown anaerobically with fumarate as the electron acceptor and DNA was extracted using a DNeasy blood and tissue kit (Qiagen, Hilden, Germany). The draft genome sequence of OR-1 was prepared using the Roche 454-GS Junior platform. The 243,751 sequencing reads for OR-1 were assembled using the Roche GS De Novo Assembler (Newbler v2.8). After removal of sequences with low sequencing depth, the resulting assembly contains 63 contigs consisting of 4,554,266 bp, with a G+C content of 54.2% and genome coverage of 23×. Genome annotation was performed using Prokka 1.9 (5), yielding a total of 4,154 protein-coding sequences (CDSs), 52 tRNA genes, and one copy of 16S-23S-5S rRNA gene. We performed BLASTp searches of protein sequences against the NCBI nr (nonredundant) database, Clusters of Orthologous Groups (COG) database (6), and Kyoto Encyclopedia of Genes and Genomes (KEGG) (7), and assigned the functional annotations of the most similar protein sequences in each database.

The genome contains two distinct arsenic islands. One consists of genes for a respiratory arsenate reductase (*arrAB*), and they are also flanked with genes for arsenic resistance including *arsR* for a regulatory protein, *acr3* for an arsenite efflux pump, *arsA* for a pump-driving ATPase, and *arsD* for a chaperone of the pump. Another island consists mainly of genes for arsenic resistance including that for a detoxifying arsenate reductase ArsC (*arsR*-*arsC*-*acr3*-*uspA*-*uspA*-ORF-*arsA*-*arsD*-ORFs-*arsD*). The draft genome contains at least 83 genes coding for *c*-type cytochromes, among which 73 are multiheme cytochromes. A set of genes for type IV pilus assembly proteins (*pil* genes) as well as for flagellar production (*fli* and *flg* genes) is identified. Genes for antioxidant proteins including superoxide dismutase, superoxide reductase, glutathione peroxidase, peroxiredoxin, glutaredoxin, and rubrerythrin are found, but the catalase-peroxidase gene is absent. In addition, genes for oxygen-metabolizing enzymes such as cytochrome *c* oxidase, cytochrome *bd*-I ubiquinol oxidase, and protoporphyrinogen oxidase are identified. A nearly complete set of genes for a reductive acetyl-CoA pathway, except for the formate-tetrahydrofolate ligase gene, is found, but there are no other autotrophic CO_2_ fixation pathway genes such as ribulose-1,5-bisphosphate carboxylase/oxygenase and ATP-citrate lyase.

### Nucleotide sequence accession numbers.

The OR-1 whole-genome shotgun project has been deposited at DDBJ/EMBL/GenBank under the accession no. BAZF00000000. The version described in this paper is the first version, BAZF01000000 (BAZF01000001 to BAZF01000063).
